# Linear and Circular Long Non-Coding RNAs in Acute Lymphoblastic Leukemia: From Pathogenesis to Classification and Treatment

**DOI:** 10.3390/ijms23084442

**Published:** 2022-04-18

**Authors:** Yasen Maimaitiyiming, Linyan Ye, Tao Yang, Wenjuan Yu, Hua Naranmandura

**Affiliations:** 1 The Affiliated Sir Run Run Shaw Hospital, and Department of Public Health, Zhejiang University School of Medicine, Hangzhou 310058, China; yasinjan@zju.edu.cn (Y.M.); 22118903@zju.edu.cn (L.Y.); 12018364@zju.edu.cn (T.Y.); 2 Cancer Center, Zhejiang University, Hangzhou 310058, China; 3 NHC and CAMS Key Laboratory of Medical Neurobiology, School of Brain Science and Brain Medicine, Zhejiang University, Hangzhou 310058, China; 4 Department of Hematology, First Affiliated Hospital, Zhejiang University School of Medicine, Hangzhou 310058, China; 5 Liangzhu Laboratory, Zhejiang University Medical Center, Hangzhou 311121, China

**Keywords:** acute lymphoblastic leukemia, ALL, long non-coding RNA, lncRNA, circular RNA, circRNA, pre-mRNA splicing, B-cell, T-cell, pathogenesis, refractory/relapsed disease, prognosis

## Abstract

The coding regions account for only a small part of the human genome, and the remaining vast majority of the regions generate large amounts of non-coding RNAs. Although non-coding RNAs do not code for any protein, they are suggested to work as either tumor suppressers or oncogenes through modulating the expression of genes and functions of proteins at transcriptional, posttranscriptional and post-translational levels. Acute Lymphoblastic Leukemia (ALL) originates from malignant transformed B/T-precursor-stage lymphoid progenitors in the bone marrow (BM). The pathogenesis of ALL is closely associated with aberrant genetic alterations that block lymphoid differentiation and drive abnormal cell proliferation as well as survival. While treatment of pediatric ALL represents a major success story in chemotherapy-based elimination of a malignancy, adult ALL remains a devastating disease with relatively poor prognosis. Thus, novel aspects in the pathogenesis and progression of ALL, especially in the adult population, need to be further explored. Accumulating evidence indicated that genetic changes alone are rarely sufficient for development of ALL. Recent advances in cytogenic and sequencing technologies revealed epigenetic alterations including that of non-coding RNAs as cooperating events in ALL etiology and progression. While the role of micro RNAs in ALL has been extensively reviewed, less attention, relatively, has been paid to other non-coding RNAs. Herein, we review the involvement of linear and circular long non-coding RNAs in the etiology, maintenance, and progression of ALL, highlighting the contribution of these non-coding RNAs in ALL classification and diagnosis, risk stratification as well as treatment.

## 1. Introduction

**Figure 1:**
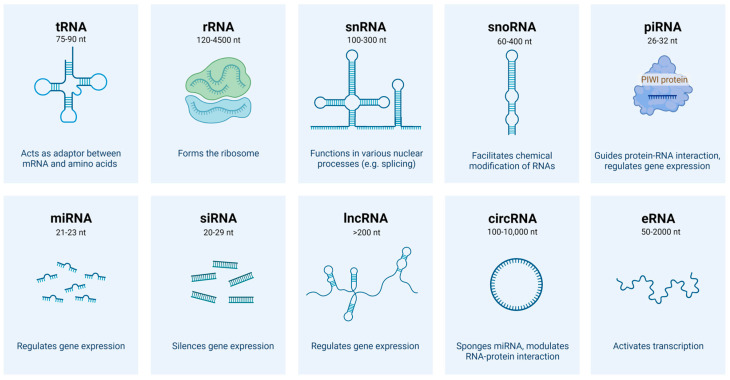
Schematic representation of major non-coding RNA types produced in cells. Size of each ncRNA type and main functions are included in the same panel as well. The figure is created with Biorender (Biorender.com (accessed on 28 January 2022)).

Ribonucleic acid (RNA) is well-known as functioning in gene transcription and translation to generate numerous proteins, which exert various biological functions. While the coding regions only account for around 1.5% of the entire human genome, the remaining non-coding regions are also extensively transcribed to produce large amount of non-coding RNAs (ncRNAs) [1,2]. The pre-mRNA splicing, processing as well as other cellular events also generate some non-coding RNAs such as circular RNAs (circRNAs) [3]. According to reports from the Encyclopedia of DNA Elements (ENCODE) project, up to 80% of the human genome has the capacity to transcribe into ncRNAs [4,5]. Based on their size and/or location and/or interacting partners, ncRNAs are classified into several types including transfer RNAs (tRNAs), ribosome RNAs (rRNAs), small nucleolar RNAs (snoRNAs), small nuclear RNAs (snRNAs), microRNAs (miRNAs), small interfering RNAs (siRNAs), long non-coding RNAs (lncRNAs), circular RNA (circRNAs), piwi-interacting RNAs (piRNAs), and enhancer RNAs (eRNAs) (Figure 1) [2,6]. Accumulating evidence indicated that different types of ncRNAs are important players in modulation of complex molecular and cellular processes [2,6,7]. Indeed, although there is a lack of open reading frames (ORFs) to code for proteins, these ncRNAs exert important functions in the transcription of genes, as well as RNA splicing, chemical modification, stability, and translation among others (Figure 1). As emerging sculptures of the epigenetic landscape, gaining comprehensive knowledge on the features and functional versatilities of these molecules in normal and cancerous conditions might help in directing new therapeutic interventions in the future.

Acute lymphoblastic leukemia (ALL) is characterized by differentiation-block at B/T-precursor stage lymphoid progenitors that can invade BM, peripheral blood, and extramedullary sites (e.g., the central nervous system) [8]. These committed cells acquire resistance to apoptosis and differentiation, and display hallmark cancer phenotypes including continuous self-renewal, abnormal proliferation, and metastasis among others. Diagnosis is established by the presence of more than 20% lymphoblasts in the BM or peripheral blood [9,10]. According to the committed cells of origin, ALL is classified into B-cell ALL (B-ALL), and T-cell ALL (T-ALL). B-ALL constitutes approximately 80–85% of total ALL cases with T-ALL comprising the rest [10]. B-ALL is further divided into many genetic subtypes according to the involved chromosomal/genetic abnormalities such as chromatin number alterations (e.g., high hyperdiploid, near haploid, low hypodiploid), rearrangements (e.g., *MLL*, *ZNF384*, *MEF2D*, *DUX4*), deletions (e.g., *ERG*), fusion oncogene producing translocations (e.g., *ETV6-RUNX1*^+^, *BCR-ABL1*^+^, *E2A-BPX1*^+^, *E2A-HLF*^+^) and so on [8,11,12]. Meanwhile, T-ALL is classified into four main subtypes according to the characteristic oncogenic aberrations, namely TLX (*TLX3* rearrangements or *HOXA* activating events), TLX1/NKX2.1 (*LTX1* or *NKX2.1* rearrangements), TAL/LMO (high expression of *TAL1*, *TAL2*, *LMO1/2/3*), and early thymocyte progenitor (ETP) /immature-ALL (high expression of *LMO2*, *LYL1*, *HHEX,* and *BCL2*) [10,13]. Such complexity of molecular and cytogenetic hallmarks determined heterogeneity of ALL, which is also reflected by the differing response of varying ALL subtypes to similar therapeutic regimens [8,14,15]. 

It is noteworthy that the incidence of ALL is highest among children aged 1–4 years and declines with aging with a slight increase after the age of 50 [8,16]. Thus, ALL represents the most frequent acute leukemia in the pediatric population, it is also the second frequent acute leukemia of adults [8,9,10]. The prognosis of ALL differs a lot among the pediatric and adult populations. With currently used state-of-the-art treatments, long-term remission of adult patients with ALL barely reaches 30–40% as compared to that of more than 80% in the pediatric ALL population [9,17]. Furthermore, relapse of ALL remains a leading cause of childhood cancer-related death [18]. Therefore, a comprehensive mechanistic understanding of the etiology and progression of ALL is of the utmost importance to develop better clinical options for management of refractory/relapsed ALL patients.

Recent developments in high-throughput sequencing technologies and bioinformatics tools have enabled comprehensive molecular characterization of various non-coding RNA expression profiles in normal and cancerous cells. Several types of ncRNAs including rRNAs, tRNAs, snRNAs, and snoRNAs are constitutively expressed in most cell types and essential for cell viability; thus, they are categorized as housekeeping ncRNAs [6,7]. Meanwhile, apart from the ncRNAs mentioned above, other types of ncRNAs such as miRNAs, lncRNAs, circRNAs, and piRNAs are classified as regulatory ncRNAs, which show tissue-specific as well as spatiotemporal expression patterns and function as tumor suppressors or oncogenes through modulating gene expression via interactions with other biomolecules (e.g., coding and non-coding RNAs, DNAs, and proteins) [6,7]. It is reported that *ETV6-RUNX1* is present in B-cell progenitors years before the onset of ALL, suggesting genetic changes alone are not sufficient to initiate ALL [19,20]. A growing body of evidence indicated the involvement of regulatory ncRNAs including miRNAs, lncRNAs, and circRNAs as cooperating events of genetic alterations in ALL [21,22], among these, the role of miRNAs in ALL pathogenesis and treatment has been extensively reviewed elsewhere [21,22,23]. In this review, we mainly recapitulate the involvement of linear and circular long non-coding RNAs in the etiology, maintenance, and progression of ALL, providing a comprehensive picture to reveal the implications of these long non-coding RNAs in ALL diagnosis, risk stratification as well as treatment.

## 2. Current Challenges in ALL Treatment

ALL is a highly heterogeneous disease. In clinic, ALL cases are stratified into favorable-, intermediate-, and adverse-risk groups based on the cytogenetic profiles [9]. Frontline treatment of ALL conventionally includes sequential phases of induction, consolidation, intensification and maintenance chemotherapy with regimens containing vincristine, cyclophosphamide, anthracyclines, L-asparaginase, and glucocorticoids among others, accompanied by central nervous system (CNS) prophylactic intrathecal chemotherapy with methotrexate alone, or methotrexate combined with cytarabine and hydrocortisone/dexamethasone, with or without CNS irradiation [8,9]. Tyrosine kinase inhibitors (TKIs, e.g., imatinib, dasatinib and ponatinib) are used in combination with the chemotherapy regimen for BCR-ABL1^+^ ALL (Ph^+^ ALL) patients [8,24]. For eligible patients (mostly MRD positive non-adolescent adult patients), allogenic stem cell transplantation (Allo-SCT) is recommended [8,25]. In addition, novel agents including monoclonal antibody drugs, proteasome inhibitors, JAK inhibitors, PI3K-mTOR inhibitors, and chimeric antigen receptor (CAR) T-cell therapy were also evaluated in clinical trials for ALL [8,26,27]. These novel agents have shown certain potential in improving ALL patient disease-free survival (DFS) when used in combination with conventional chemotherapy [8,24,25,26,27]. Nevertheless, obtaining long-term remission/cure in relapsed or refractory ALL patients is a current need.

Treatment of pediatric ALL represents a major success story and paradigm of malignant cancer elimination by dose-intensified chemotherapy regimens [28]. This is attributable to the improved risk stratification based on genetics as well as clinical characteristics at diagnosis, optimized chemotherapy regimens according to early treatment response as measured by minimal residual disease (MRD), and better tolerance of children to the chemotherapy regimens than adults [8,9,28,29]. However, relapsed ALL is still the second leading cause of childhood cancer-related death [18]. In addition, more than 50% of adult ALL patients who achieved a complete remission will ultimately relapse, and about 10% of adult ALL patients are initially refractory to the induction chemotherapy [30]. B-ALL subtypes associated with low hypodiploid (32–39 chromosomes), BCR-ABL^+^, and MLL (KMT2A) rearrangements, as well as T-ALL subtypes associated with HOXA activating events, mutations in IL-7R signaling molecules (IL7R, JAK1/3, STAT5B, N/KRAS, AKT), PTEN-inactivating events, and DNMT3A mutations among others develop refractory disease and show very poor prognosis [8,10,13,31]. There are few effective therapeutic approaches for relapsed/refractory ALL. One exception is the Ph^+^ ALL (BCR-ABL1^+^ ALL), which was once the most aggressive genetic abnormality in ALL and displayed very poor prognosis for both children and adults [32]. With the development and application of BCR-ABL1-targeted TKIs, the DFS of Ph^+^ ALL patients increased from around 20% to more than 60%, but there is still huge room for improvement [24,33]. Therefore, constant updates in therapeutic options are required to improve the survival rate of ALL patients.

CNS is the major site for the extramedullary invasion of most acute leukemias including ALL and the survival rate of CNS-infiltrated leukemia is rather poor [34,35]. In the early era of ALL treatment, CNS-targeted therapy was not included in the regimen, leading to as high as 80% of CNS relapse in all patients [36,37]. Later on, CNS prophylactic intrathecal chemotherapy with the ability to penetrate the blood–brain barrier was introduced into ALL treatment and largely reduced the overall risk of CNS relapse to 5% [38]. Notably, around 30–40% of relapsed ALL patients show CNS involvement, albeit many of them achieve a second complete remission (CR) [39]. The CNS-infiltrated ALL patients showing resistance to intrathecal chemotherapy respond well to the intracranial irradiation but face substantial neurotoxicity risks [40]. Common risk factors of CNS infiltration in ALL includes age, T-cell lineage (T-ALL), hyperleukocytosis, and some specific chromosomal aberrations such as MLL rearrangement, BCR-ABL1, and E2A-BPX1 fusion genes etc. [41]. In contrast to the role of qPCR-based MRD detection for monitoring BM responses and guiding risk stratification-based treatment adjustments [29,42], feasible and facile strategies for the detailed evaluation of CNS response are still lacking [43]. Thus, identification and comprehensive characterization of relapse (including CNS relapse) or refractory prone clones might offer precious opportunities to improve treatment outcomes in ALL patients.

## 3. Biogenesis and Biological Functions of lncRNAs

LncRNAs are characterized as ncRNA species of a size greater than 200 nucleotides (nt) without significant protein-coding capacity, which are yielded from eukaryotic RNA transcription and processing events [6,7]. This lenient definition determines lncRNAs as a large and highly heterogeneous family of transcripts with differing biogenesis pathways and genomic origins [44]. According to the estimates from ENCODE Project Consortium (GENCODE release 23), the human genome encodes more than 28,000 different lncRNAs [45]. Many lncRNAs display tissue-specific and spatiotemporal expression patterns, suggesting their important roles in cell fate determination. For instance, a previous study found that lncRNA X-inactive-specific transcript (Xist) knockout mice die early in embryogenesis as a result of two active X chromosomes induced extra-embryonic phenotype [46], highlighting the importance of lncRNAs in physio-pathological processes. Such distinct expression patterns and crucial functions of lncRNAs confers on them the potential of being used as feasible disease biomarkers as well as therapeutic targets.

**Figure 2:**
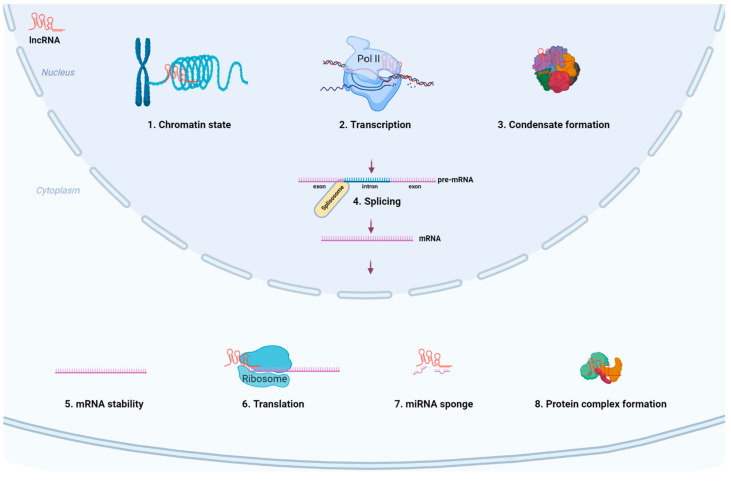
Main biological functions of lncRNAs in cells. In general, lncRNAs with multiple exons are exported to the cytoplasm similar to mRNAs, while intronic lncRNAs are retained in the nucleus. Due to the flexible structure and long size, lncRNAs interact with DNA, RNA, and protein, thereby regulating chromatin state (histone modification, DNA methylation), transcription, pre-mRNA stability as well as splicing and processing, nuclear condensate formation (i.e., paraspeckles, nuclear speckles), mRNA stability, translation, and sponging miRNAs as well as orchestrating protein complex formation. The figure is created with Biorender (Biorender.com (accessed on 28 January 2022)).

Genomic origins of lncRNAs include promoter upstream regions, enhancers, intergenic/intragenic regions, and opposite strand of protein coding genes, among others [7,44]. LncRNAs are mostly transcribed by RNA polymerase II (Pol II), and less frequently by other RNA polymerases [44,47]. Thus, the resulting lncRNAs are often capped by 7-methyl guanosine (m^7^G) at their 5′ ends, polyadenylated at their 3′ ends, and even spliced similarly to mRNAs [44,48]. In addition, a considerable amount of lncRNAs are generated from long primary transcripts though other non-canonical RNA stabilizing pathways, such as RNase P cleavage to generate a mature 3′ end and capping by snoRNA-protein complexes at both ends or the 5′ end [49,50,51]. Taking advantage of flexible structure, lncRNAs interact with DNA, RNA, and proteins, thereby regulating gene expression and other bio-processes via affecting the chromatin state, transcription, biomolecular condensate formation, pre-mRNA splicing, mRNA stability, and translation (Figure 2) [44,45,48]. For instance, lncRNA ANRIL mediates polycomb repressive complex 1 (PRC1) and PRC2 recruitment to the promoters of its adjacent CDKN1B and CDKN2A genes, thereby controlling their expression and regulating cell senescence [52,53]. The lncRNA Xist inactivates a large proportion of the genes on the X chromosome via a complex interplay of protein interactors [54,55,56]. Recently, Statello et al. described various roles of lncRNAs in detail [2]. Together, these findings suggest that lncRNAs are feasible targets to intervene gene expression.

As compared to mRNAs, Pol II-transcribed lncRNAs are less efficiently processed, and a large proportion of such lncRNAs are retained in the nucleus [57,58]. These lncRNAs are either tethered to chromatin or participate in the formation of biomolecular condensates (BioMCs, also known as membranelles organelles) in the nucleus (Figure 2). For example, the long isoform of the lncRNA nuclear paraspeckle assembly transcript 1 (NEAT1) contributes to the organization and functions of paraspeckles via recruiting core proteins NONO and SFPQ to orchestrate the assembly of paraspeckles through liquid–liquid phase separation, regulating gene expression by sequestrating proteins or mRNAs with inverted repeats in their 3′ UTRs [59]. LncRNA metastasis-associated lung adenocarcinoma transcript 1 (MALAT1) participates in the formation of nuclear speckles, regulating transcription and pre-mRNA splicing [60,61]. It is estimated that total RNA concentration in the nucleus is much higher than that in the cytoplasm [62], which is likely caused by, at least in part, the large amount of nuclear-retained lncRNAs. This characteristic high RNA concentration is reported to prevent aggregation of prion-like RNA binding proteins (RBPs) such as FUS and TDP43 in the nucleus [62,63], while FUS and TDP43 tend to aggregate in the cytoplasm probably due to less RNA concentration, causing neurodegenerative diseases including frontotemporal dementia and amyotrophic lateral sclerosis [62,64]. Interestingly, some lncRNAs with multiple exons are transported to the cytoplasm via nuclear RNA export factor 1 (NXF1) dependent pathway, interacting with RBPs and regulating mRNA stability as well as translation [48,65]. Accumulating evidence indicated that lncRNAs are widely associated with a majority of cancer types [66,67,68]. The aberrant expression of these lncRNA transcripts has been implicated in tumorigenesis, metastasis, cancer stage progression, and patient survival [68,69,70]. Importantly, these lncRNA molecules are easily targetable by antisense oligonucleotides (ASOs, suitable for downregulating nuclear lncRNAs) or siRNAs [48,71,72], suggesting that lncRNAs can be a class of druggable vulnerability in cancer.

## 4. LncRNAs in ALL Classification, Diagnosis, Pathogenesis, and Resistance to Treatment

Several studies have indicated that lncRNAs are associated with hematopoietic development [73,74,75,76], implying their potential involvement in hematological malignancies. Indeed, as important players in epigenetic regulation, lncRNAs have been demonstrated to function in various aspects of ALL (Table 1, Table 2 and Table 3) [77,78,79,80,81,82,83,84,85,86,87,88,89,90,91,92,93,94,95,96,97,98,99,100,101,102,103,104,105,106,107,108,109,110,111,112,113,114,115,116], which are summarized in this part.

### 4.1. LncRNAs in ALL Classification and Diagnosis

According to a study by Melo et al., the lncRNA expression profile showed significant alterations between different leukemia types and different subtypes of the same leukemia including ALL [117]. Another study found T-ALL subtype-specific lncRNA expression profiles, suggesting the involvement of lncRNAs as cooperators or downstream effectors during the corresponding oncogene-mediated leukemogenesis [118]. Alterations in chromatin and DNA modification also affect lncRNA expression profiles. It is reported that lncRNAs methylation per base pair shows a distinct profile between ALL and normal B-cell lymphoid precursors as well as among three sub clusters of ALL cases [119]. In human B-ALL samples, it was found that the lncRNA expression profile also correlates with cytogenetic abnormalities [102]. Likewise, many other studies also revealed subtype-specific and/or relapse-specific lncRNA signatures in ALL patent samples [77,120,121,122,123,124,125,126,127,128,129,130,131,132,133,134]. These studies suggest that lncRNAs are associated with the pathogenesis of ALL and might be used as molecular markers for disease classification and diagnosis.

### 4.2. LncRNAs as Cooperating Events of Genetic Abnormalities in T-ALL Pathogenesis

T-ALL patients have a worse prognosis than those with B-ALL probably due to the less-known and more complex mechanism on its etiology, maintenance, and progression [8,27], suggesting the importance of exploring novel aspects in the pathogenesis and progression of T-ALL. Zhang et al. found that a lncRNA named T-ALL-related long non-coding RNA (T-ALL-R-LncR1) is markedly expressed in T-ALL Jurkat cells and 50% of T-ALL patient primary cells [79]. Importantly, knockdown of T-ALL-R-LncR1 led to apoptosis in Jurkat cells [79]. This finding suggests that the lncRNA T-ALL-R-LncR1 confers resistance to apoptosis, playing an oncogenic role in T-ALL development. It is well demonstrated that T cell acute lymphocytic leukemia protein 1 (TAL1, also known as SCL) is one of the most prevalent oncogenic transcription factors in T-ALL [10,13]. Generally, TAL1 coordinates with other transcription factors including GATA3, RUNX1, and MYB to regulate the expression of their downstream target genes in T-ALL cells [135]. Interestingly, TAL1 reportedly activated a subset of lncRNAs, some of which are regulated by GATA3,RUNX1, and MYB in a coordinated manner [133]; another subset of lncRNAs negatively regulated by TAL1 were also identified. Notably, the transcription factors T-cell leukemia homeobox 1 (TLX1) and TLX3 are key drivers of the TLX1/NKX2.1 and TLX subgroups of T-ALL, respectively [10,43]. A study showed that TLX1 directly regulates a set of lncRNAs, some of which are marked by super-enhancers [134], indicating the involvement of lncRNAs in TLX1-induced gene expression regulation. Intriguingly, some lncRNAs give rise to other ncRNA types following further processing. It was reported that TLX3 binds and transactivates lncRNA LINC00478, which is the host of miR125-b, to regulate miR-125b production, through which supports growth and invasiveness of T-ALL cells [81]. Collectively, these findings suggest that lncRNAs are crucial downstream effectors of major genetic abnormalities in the pathogenesis of T-ALL.

T-ALL is driven by oncogenic transcription factors (e.g., LTX1, LTX3 or HOXA, and TAL/LMO) that act along with secondary acquired mutations [10,13]. For instance, NOTCH1 activating mutations are identified in more than 50% of T-ALL cases, suggesting a key role in driving the disease [136]. A study by Durinck et al. established a novel lncRNA network that acts downstream of NOTCH1 during normal and malignant thymocyte development [137], specifically, they identified 40 lncRNAs that are positively regulated by NOTCH1 in both normal and malignant T lymphocytes, supporting an important role for these lncRNAs as downstream effectors in NOTCH1-regulated T-cell biology. Another study also uncovered a set of T-ALL-specific lncRNAs, many of which are directly regulated by the NOTCH1/RPBJκ activator complex [77]; the authors showed that one specific NOTCH-regulated lncRNA, LUNAR1, is required for efficient T-ALL growth in vitro and in vivo due to its ability to enhance insulin-like growth factor 1 (IGF1) receptor (IGF1R) mRNA level and sustain IGF1 signaling. Likewise, a lncRNA named NALT, which is located only 100 bp away from NOTCH1 gene, was upregulated in human NOTCH1-activated T-ALL samples [80]; increased expression of NALT dramatically promoted cell proliferation, while knockdown of NALT caused the opposite effect; mechanistically, nuclear located NALT functioned as a transcription activator causing activation of the NOTCH1 signaling pathway. Specific lncRNAs and their effects in T-ALL pathogenesis are summarized in Table 1.

### 4.3. LncRNAs as Cooperating Events of Genetic Abnormalities in B-ALL Pathogenesis

Fusion genes produced by chromosomal translocations are often strong oncogenic drivers and cytogenetic abnormalities in hematological malignancies such as acute myeloid leukemia (AML) or lymphoma [34,138]. Similarly, fusion oncogenes represent a prominent class of oncogenic drivers in ALL as well [8,9,139]. ETV6-RUNX1 generated by t(12;21) is the most common oncogenic fusion gene in childhood B-ALL [140]. A comprehensive analysis of the lncRNA transcriptome in ETV6-RUNX1^+^ B-ALL revealed the fusion protein-specific lncRNA expression signature [141]. Further analysis showed that expression of lncRNAs NKX2-3-1, TIMM21-5, ASTN1-1, and RTN4R-1 are linked to the oncogenic fusion protein. Knockdown of NKX2-3-1 and RTN4R-1 in ETV6-RUNX1^+^ cells reversed the expression of genes deregulated by the ETV6-RUNX1 fusion protein [141]. Likewise, lncRNA CASC15 is overexpressed in ETV6-RUNX1^+^ B-ALL, and shows increased expression of its chromosomally adjacent gene, SOX4, which encodes a transcriptional activator in lymphocytes, thereby upregulating cell survival, and proliferation [96]. An oncogenic lncRNA TCL6 was also upregulated in the ETV6-RUNX1^+^ B-ALL and probably associates with poor disease-free survival [123]. Collectively, these findings suggest the involvement of lncRNAs in fusion oncogene-mediated pathogenesis and progression of ALL.

Fusion of the mixed lineage leukemia (MLL) gene with other partner genes are frequent in pediatric leukemias, and MLL-rearranged B-ALL has long been considered a refractory disease due to the poor prognosis of associated patients [142]. A genome-wide lncRNA expression study found 52 upregulated and 59 downregulated lncRNAs between MLL-rearranged and MLL-unarranged ALL patient samples [143]; bioinformatics analysis showed that several lncRNAs correspond to the expression of the MLL fusion protein partner genes, such as HOXA and MEIS1 among others, and some other lncRNAs are associated with histone-related functions or membrane proteins; further, three subtypes of MLL rearranged ALL (MLL-AF4^+^, MLL-AF9^+^, and MLL-ENL^+^) showed a translocation specific lncNRA expression signature [143]. Another study reported that lncRNA BALR-6 is highly expressed in MLL-rearranged patient samples [98]; knockdown of BALR-6 reduced cell proliferation and induced apoptosis, while overexpression of BALR-6 caused a significant increase in early hematopoietic progenitor populations in murine BM transplantation experiments, suggesting that its dysregulation may cause developmental changes. Based on thorough literature searching, specific lncRNAs and their effects in B-ALL pathogenesis are summarized in Table 2.

### 4.4. LncRNAs and Immune Response Modulation in ALL Pathogenesis and Treatment

The immune suppressive tumor microenvironment is proven essential in the pathogenesis of many cancer types [144,145,146]. LncRNAs NONHSAT027612.2 and NONHSAT134556.2 were significantly elevated in the blood and BM of pediatric ALL patients, and may serve important roles in the pathogenesis of childhood ALL via suppressing immune response-associated pathways [125]. Besides, in the BM of patients with childhood T-ALL, membrane-localized and cytoplasm-localized lncRNA insulin receptor precursor (INSR) promoted CD4^+^ regulatory T-cell distribution and decreased the percentage of CD8^+^ cytotoxic T-cells in the BM of pediatric T-ALL patients, facilitating leukemic cell growth via immune suppression [147]. Mechanistically, through direct binding with INSR protein, lncRNA-INSR blocked the INSR ubiquitination site, causing abnormal accumulation and activation of INSR and the PI3K/AKT-signaling pathway. LncRNAs are also implicated in CART therapy of B-ALL [148]; specifically, a set of lncRNAs showed high degree of co-expression with transcription factors or histones (i.e., FOS and HIST1H4B) and were associated with immune processes during CAR-T therapy of B-ALL. These findings suggest that lncRNAs are important players in the immune response regulation in ALL pathogenesis and treatment.

### 4.5. LncRNAs in Susceptibility, Treatment Response and Prognosis of ALL

Single nucleotide polymorphism (SNP) is common in the human genome, and has profound implications in the pathogenesis/susceptibility of varying diseases. It was reported that the rs2147578 polymorphism of lncRNA LAMC2-1 may be a risk factor for developing childhood ALL, suggesting that SNPs might confer oncogenic properties on lncRNAs [149]. LncRNA PAX8-AS1 is located in the upstream region of PAX8, a gene encodes the transcription factor PAX8 required for cell growth and differentiation during embryonic development, and potentially regulates PAX8 expression [150]. Similarly, Bahari et al. reported that polymorphisms rs4848320 and rs6726151 of PAX8-AS1 might be risk factors for the development of childhood ALL [151]. Similarly, rs7158663 AG/AA genotypes of lncRNA MEG3 were associated with higher susceptibility to childhood ALL [152]. These findings indicate that some SNPs on lncRNAs are indicative of higher susceptibility for developing ALL.

Fernando et al. reported a correlation of high lncRNA BALR-2 expression with the diminished response to prednisone treatment and poor survival in patients [103]. BALR-2 knockdown led to reduced proliferation, increased apoptosis, as well as augmented sensitivity to prednisone treatment via activation of the glucocorticoid (GC) response pathway in both human and mouse B cells [103]. Another study reported that five lncRNAs are specifically upregulated in childhood B-ALL, and these lncRNAs had significant impacts on cell proliferation, migration, apoptosis, and treatment response [95]. In particular, expression of lncRNA RP11-137H2.4 was negatively associated with GC response [95]. Similarly, lncRNA GAS5 was also found to be a potential marker of GC response in remission induction therapy of childhood ALL [153]. A high expression of the lncRNA CDKN2B-AS1 level was associated with Adriamycin resistance [83]. Gioia et al. reported that LncRNAs RP11-624C23.1 and RP11-203E8 were downregulated in ALL, and restoring their expression in ALL cells’ increased sensitivity to genotoxic stress (e.g., chemotherapy agents), possibly by modulating the DNA damage response pathway [154]. These studies suggest that lncRNAs are important indicators of treatment response in ALL therapy.

Due to the strong oncogenic roles, some lncRNAs might predict prognosis in ALL patients. For example, lncRNA ZEB1-AS1 promoted the activation of IL-11/STAT3 signaling pathway by interacting with IL-11 in B-ALL cells, and a high expression of lncRNA ZEB1-AS1 predicted a poor prognosis for B-ALL patients [96]. A bioinformatics analysis presented a lncRNA-mRNA-based classifier that might be clinically useful to predict the recurrence and prognosis for childhood ALL [155]. It is also reported that the lncRNAs NEAT1 and MALAT1 sponges miR-335-3p, increasing the multidrug-resistance gene ATP-binding cassette sub-family A member 3 (ABCA3) expression and leading to poor prognosis in childhood ALL [156]. Many other studies also found similar roles of lncRNAs in prediction of the prognosis in ALL patients [78,106,113,140,151,157,158,159].

In conclusion, alterations in the expression of lncRNAs are important cooperating events in ALL pathogenesis, maintenance, and progression. Therefore, lncRNAs could be applied in diagnosis, classification, risk stratification, prognosis, as well as treatment of ALL.

## 5. Biogenesis and Biological Functions of Circular RNAs

**Figure 3:**
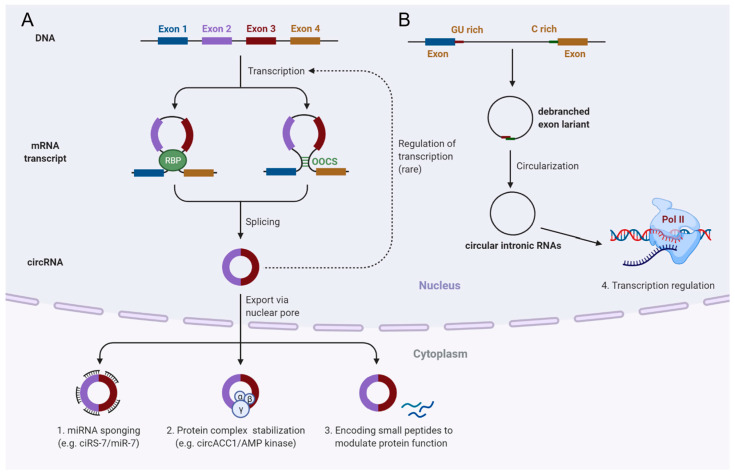
Main biogenesis pathways and functions of circRNAs in cells. (**A**), Biogenesis of exonic circRNA from back-splicing of pre-mRNA. RNA-binding proteins (RBPs) or orientation-opposite complementary sequences (OOCS) that form loop-like structures facilitate back-splicing and circRNAs formation; these circRNAs are generally exported to the cytoplasm and act as a miRNA sponge, protein complex coordinators as well as small peptide producers to modulate protein function; (**B**), Biogenesis of lncRNAs from intron lariats. A consensus RNA motif containing a 7-nt GU-rich element near the 5′ splice site and an 11-nt C-rich element near the branch point prevents detaching of the intron lariats and promotes intronic circRNA formation; these circRNAs are retained in the nucleus and mainly regulate Pol II-mediated transcription. The figure is created with Biorender (Biorender.com (accessed on 28 January 2022)).

Circular RNAs (circRNAs) represent a class of emerging ncRNA type, which are discovered by non-polyadenylated RNA-seq [50,160]. Similar to the lncRNAs, circRNAs are also transcribed by Pol II and have a wide range of size (100–10,000 nt) [160]. CircRNAs are the circularization products from precursor RNA splicing events, which can be generated from exons, introns, intergenic regions, untranslated regions (UTRs), or even tRNAs [160,161]. Generally, two major groups of circRNAs are generated from the Pol II-transcribed RNA precursors following splicing events [44,152,153,154,155,156,157,158,159,160,161,162,163,164]. On the one hand, back-splicing events of exons lead to connection of a downstream 5′ splice site with an upstream 3′ splice site to yield a group of circRNAs (Figure 3A) [162,163]. Orientation-opposite complementary sequences (OOCS), that juxtapose flanking introns of circularized exons to form RNA pairs or RNA binding proteins (RBPs) that bind to pre-mRNAs to connect flanking introns, generally facilitate back-splicing and circRNAs formation (Figure 3A) [162,163]. On the other hand, another group of circRNAs are generated from spliced intron lariats (Figure 3B) [164]; specifically, when the spliced intron lariats fail to debranch, covalent 2′,5′-phosphodiester bonds form to circularize these lariats, thereby generating circRNAs (known as intronic circRNAs) (Figure 3B) [164]. Accumulating evidence indicated that intronic circRNA formation depended on a consensus RNA motif containing a 7-nt GU-rich element near the 5′ splice site and an 11-nt C-rich element near the branch point (Figure 3B) [44,164]. Accordingly, circRNAs are characterized by a covalently closed loop structure, lacking both 5′- and 3′-ends, polyadenylated tail, and a 5′-CAP [160,161,162,163,164]. This unique structure confers exonuclease digestion resistance to circRNAs, largely enhancing their stability.

In comparison with mRNAs, circRNA production from pre-mRNA back-splicing is relatively slow, leading to a lower production rate of circRNAs than their linear mRNA counterparts [165]. CircRNAs containing one or more exons are exported to the cytoplasm [166]. These cytoplasmic circRNAs exert various biological functions such as miRNA sponges to regulate gene expression, protein complex coordinators, and others (Figure 3A). Meanwhile, the intronic circRNAs are retained in the nucleus and mainly regulate Pol II-mediated transcription (Figure 3B). In addition, accumulating evidence also indicated that some cytoplasmic circRNAs are transcribed into small peptides, thereby regulating protein–protein interactions [167]. Interestingly, the detected levels of some circRNAs are higher than their counterpart mRNAs, possibly due to their resistance to RNase clearance and intracellular accumulation [168]. The high stability of circRNAs also make them ideal biomarkers for liquid biopsy [169]. Collectively, circRNAs are important epigenetic players with various biological functions.

CircRNAs are implicated in cellular differentiation and maintenance of stem cell pluripotency [170,171]. For instance, a study found that the expression of circRNAs is cell type-specific and increases upon differentiation [170]. CircBIRC6 sponges inhibitory miRNAs such as miR-34a and miR-145, thereby activates multiple stemness markers including NANOG, OCT4, and SOX2, maintaining stem cell pluripotency [171]. These findings suggest that circRNAs might have pathological roles in cancer. Indeed, a large body of evidence demonstrated that circRNAs regulate hallmark phenotypes of cancer, such as angiogenesis, immune suppression, proliferation, cell cycle progression, apoptosis, invasion, and metastasis among others in many cancer types [161,172]. Therefore, circRNAs also represent a group of ncRNAs that is targetable in the fight against cancer.

## 6. CircRNAs in ALL Classification, Pathogenesis and Treatment

Owing to the unique circular structure, circRNAs display higher stability than other ncRNA types [169]. Similar to their role in other malignancies, circRNAs are also implicated in ALL classification, pathogenesis, and treatment, which will be recapitulated in detail in this part.

### 6.1. CircRNAs in ALL Classification and Diagnosis

Precise classification of ALL is instrumental for risk stratification and selection of optimal treatment strategies [8,9,10,173,174]. Thus, uncovering the circRNA signature of cytogenetic ALL subtypes is of critical importance in improving treatment outcomes. A study revealed specific circRNAs in B-cell (e.g., circPAX5, circAFF3, circIL4R, and circSETBP1) and T-cell (e.g., circIKZF1, circTNIK, circTXK, and circFBXW7) populations [175]. They also found upregulation of circPAX5, circPVT1, and circHIPK3 in pediatric B-ALL, and disclosed circRNAs with variable expression patterns across different cytogenetic subtypes of B-ALL; specifically, circAFF2 expression level was high in TCF3-PBX1^+^ B-ALL and, to a lesser extent, in ETV6-RUNX1^+^ B-ALL; intronic circBCL2 was upregulated in ETV6-RUNX1^+^ ALL; circSETBP1 and intergenic circX were both largely reduced in MLL rearranged B-ALL; circIKZF1 was lower in BCR-ABL^+^ and hyperdiploid B-ALL than the ETV6-RUNX1^+^ subtype which had similar expression with normal B-cells [175]. Likewise, another study investigated the circRNA signature of T-ALL [175]. According to their results, circZNF609, circKPNA5, circPSEN1, and circCEP70 were upregulated in ETP-ALL; circTASP1, circZBTB44, and circBACH1 were upregulated in TLX3-rearranged T-ALL; circHACD1, and circSTAM were upregulated in HOXA-rearranged T-ALL; circCAMSAP1 was upregulated in TLX1-rearranged T-ALL; and circCASC15 was upregulated in TAL/LMO subtype of T-ALL [176]. In addition, differences in the whole circRNAs’ expression signature, as well as specific circRNA expression, reportedly differentiated ALL from AML [177]. Collectively, these findings suggest that circRNAs could be applied in the classification, risk stratification, and even diagnosis of ALL.

### 6.2. CircRNAs Acting as Oncogenes in ALL Pathogenesis

Circular RNA PVT1 (circPVT1) was first identified as a suppressor of cellular senescence [178]. It was also demonstrated to be a proliferative factor and prognostic marker in gastric cancer [179]. Subsequently, Hu et al. reported that circPVT1 is also highly expressed in ALL patient samples and cell lines [180]; knockdown of circPVT1 in ALL cells resulted in proliferation inhibition and apoptosis induction via suppression of its neighboring gene c-Myc, and antiapoptotic Bcl-2 protein expression, suggesting an oncogenic role for this circRNA in ALL. Upregulation and oncogenic properties of circPVT1 in ALL were validated in other studies as well. For instance, the circPVT1 activated NF-κB signaling pathway through reducing miR-125b, thereby promoting ALL cell proliferation and migration [181]; this group also validated that circPVT1 sequesters the tumor suppressing miR-30e to increase DLL4 expression, thereby activating NOTCH signaling and promoting T-ALL cell proliferation [182]. In addition, it was demonstrated that upregulation of circ-0000745 in ALL cell lines could enhance the cell viability through the activation of ERK pathway [183]. According to another study, circ-0000745 was overexpressed in T-ALL patient BM and/or T-ALL cell lines, and circ-0000745 predominantly regulated NOTCH1 expression via sponging miR-193b-3p, contributing to the disease pathogenesis [184]. A recent study also demonstrated that circ-0000745 contributes to ALL development partly by sponging miR-494-3p to induce NET1 expression [185]. The oncogenic circPRKCI was upregulated in T-ALL patient samples and sequestered miR-20a-5p via sponging, thereby promoting viability and inhibiting apoptosis of T-ALL cells [186]. Circ-PRKDC was also upregulated in T-ALL patient samples as well as cell lines, and sponged miR-653-5p to depress RELN expression, leading to activation of the PI3K/AKT/mTOR signaling pathway and promotion of cell proliferation, as well as inhibition of apoptosis [187]. Collectively, these findings suggest that certain oncogenic circRNAs are upregulated in ALL and contribute to the pathogenesis of disease, and these circRNAs are potential therapeutic targets for the diagnosis and treatment of ALL.

### 6.3. CircRNAs Acting as Tumor Suppressors in ALL Pathogenesis

On the other hand, circRNAs also exert tumor suppressor functions. It was reported that the cytoplasm-localized circ_0000094 expression is markedly reduced in T-ALL [188]; intriguingly, exogenous compensation of circ_0000094 inhibited T-ALL cell proliferation, migration, and invasion, and enhanced apoptosis; while knockdown of circ_0000094 showed the opposite effect. Further analysis revealed that circ_0000094 is a molecular sponge for miR-223-3p, and it could up-regulate the expression of tumor suppressor FBW7 via sequestering miR-223-3p [188]. Expression of circ_0000143 was also significantly reduced in T-ALL patient samples compared to healthy controls; overexpression of circ_0000143 inhibited T-ALL cell viability, migration and invasion, and induced apoptosis. Mechanistically, circ_0000143 acted as a molecular sponge to sequester miR-142-3p, thereby upregulating miR142-3p repressed glucocorticoid receptor α (GRα) expression [189]. Likewise, circADD2 was downregulated in ALL patient samples and cell lines [190]; overexpression of circADD2 inhibited cell proliferation and promoted apoptosis; while, knockdown of circADD2 showed the opposite effect; mechanistically, circADD2 could directly sponge miR-149-5p, a positive regulator of AKT2, leading to reduction of AKT2 and pAKT2 level [190]. These findings suggest that tumor suppressing circRNAs are downregulated in ALL, and increasing expression of these circRNAs represents a novel strategy in ALL treatment as well.

Taken together, circRNAs play carcinogenic or anticancer effects in ALL pathogenesis and progression. Their high stability could provide a unique advantage in liquid biopsy-based diagnosis. More importantly, RNA interference-based strategies or manipulation of endogenous promoters by CRISPR/Cas9-based strategies are feasible approaches to decrease oncogenic or increase tumor suppressing circRNAs expression [191]. Thus, circRNAs could be applied in diagnosis, classification, and treatment of ALL.

## 7. Conclusions

Over the last decade, tremendous progress has been made in the treatment of ALL using evidence-based, risk-adapted, MRD-guided, and multiagent chemotherapy [8,9,10,29]. Nevertheless, disease relapse still occurs for many adult patients [10,30,39]. Personalized medicine targeting the initiation, maintenance, progression, and resistance mechanisms is a promising strategy to increase survival in this set of patients [17,39]. However, although most of the genetic abnormalities resulting in ALL onset and progression are clearly defined, limited targeted therapeutics are available in clinic [10,18,37,192]. This is probably due to the low translational value of some therapeutic targets or unexpected adverse events. Thus, there is an urgent need to excavate novel therapeutic targets. As important cooperating events of genetic changes in ALL etiology, maintenance, and progression, lncRNAs and circRNAs are proven as novel classes of targetable vulnerabilities in ALL. Especially for those lnc/circRNAs specifically upregulated in certain ALL subtypes, it is facile to design and produce siRNAs or ASOs or CRISPR/Cas based tools that target and downregulate their level [190]. These comprehensive analysis and insights could facilitate the bridging of basic research to the clinical practice, promoting emergence of new therapeutic strategies to improve ALL patient outcomes.

## Figures and Tables

**Table 1:** List of lncRNAs implicated in T-ALL pathogenesis and treatment.

lncRNA	Subject	Function	Alteration	Target Regulation	Effect on All Cell Phenotype	References
*LUNAR1*	T-ALL cell lines and patient samples	oncogene	upregulated	enhancing IGF1R mRNA expression, sustaining IGF1 signaling	promoting cell growth via acting as NOTCH effector	[77,78]
*T-ALL-R-LncR1*	T-ALL cell lines and patient samples	oncogene	upregulated	inhibiting pro-apoptotic factor Par-4/THAP1 protein complex	inhibiting apoptosis	[79]
*NALT1*	T-ALL cell lines and patient samples	oncogene	upregulated	causing transcriptional activation of NOTCH1 signaling	promoting cell proliferation	[80]
*LINC00478*	T-ALL cell lines and patient samples	oncogene	upregulated	upregulating miR-125b production	promoting cell growth and invasiveness	[81]
*ARIEL*	T-ALL cell lines and patient samples	oncogene	upregulated	enhancing *ARID5B* gene expression	promoting cell growth and survival	[82]
*CDKN2B-AS1*	T-ALL cell lines and patient samples	oncogene	upregulated	sponging miR-335-3p to upregulate *TRAF5*	promoting cell proliferation and cell cycle progression	[83]
*LINC00511*	T-ALL cell lines and patient samples	oncogene	upregulated	sponging miR-195-5p to upregulate *LRRK1*	boosting cell proliferation and invasion, inhibiting apoptosis	[84]
*ANRIL*	T-ALL cell lines and patient samples	oncogene	upregulated	suppressing miR-7-5p to upregulate *TCF4*	promoting cell viability, migration, and invasion	[85]
*AWPPH*	T-ALL cell lines and patient samples	oncogene	upregulated	upregulation of *ROCK2* expression	promoting proliferation and inhibiting apoptosis	[86]
*NEAT1*	T-ALL cell lines and patient samples	oncogene	upregulated	sponging miR-146b-5p to upregulate expression of *NOTCH1*	promoting cell proliferation and growth	[87]
*H19*	T-ALL cell lines and patient samples	oncogene	upregulated	inducing SOX2, OCT-4, and NANOG expression; decreasing miR-326 level to upregulate BCL-2 expression	maintaining stemness and promoting cell proliferation; inhibiting apoptosis	[88,89,90]
*LINC00853*	T-ALL cell lines and patient samples	tumor suppressor	downregulated	upregulating *CCR9* expression	inhibiting cell proliferation, migration, and invasion	[91]
*LNC00221*	T-ALL cell lines Jurkat, CCRF-CEM, CEM/C1	tumor suppressor	downregulated	sponging miR-152-3p to upregulate *ATPA2A*	inhibiting cell proliferation and apoptosis	[92]
*VPS9D1-AS1*	T-ALL cell lines Molt-3 and Molt-4	oncogene	upregulated	inhibiting miR-419-5p, miR-214-3p to upregulate *GPX1*	inhibiting apoptosis and promoting survival	[93]

Abbreviations: *LUNAR1*, leukemia-induced noncoding activator RNA-1; *T-ALL-R-LncR1*, T-ALL-related long non-coding RNA; *NALT*, NOTCH1-associated lncRNA in T-cell acute lymphoblastic leukemia; *LINC00478*, long intergenic non-protein coding RNA 478; *ARIEL*, *ARID5B*-inducing enhancer associated long noncoding RNA; *CDKN2B-AS1*, *CDKN2B* antisense RNA 1; *LINC00511*, long intergenic non-protein coding RNA 511; *ANRIL*, antisense non-coding RNA in the INK4 locus; *AWPPH*, associated with poor prognosis of hepatocellular carcinoma; *NEAT1*, nuclear paraspeckle assembly transcript 1; *LINC00853*, long intergenic non-protein coding RNA 583; *LNC00221*, long intergenic non-protein coding RNA 211; *VPS9D1-AS1*, *VPS9D1* antisense RNA 1.

**Table 2:** List of lncRNAs implicated in B-ALL pathogenesis and treatment.

lncRNA	Subject	Function	Alteration	Target Regulation	Effect on ALL Cell Phenotype	Reference
*RP11-137H2.4*	B-ALL cell lines and patient samples	oncogene	upregulated	modulated expression of NRAS/BRAF/NF-κB MAPK cascade and cell cycle pathways	enhancing cell proliferation, migration, and apoptosis, inducing GC resistance	[94]
*CASC15*	*RUNX1/AML*^+^ B-ALL cell lines and patient samples	oncogene	upregulated	enhancing YY1-mediated regulation of the SOX4 promoter	promoting cell proliferation and survival	[95]
*ZEB1-AS1*	B-ALL cell lines and patient samples	oncogene	upregulated	binding to *IL-11* and promoting *IL-11* stability, activating STAT3	increasing cell proliferation	[96]
*BALR-6*	B-ALL cell lines and patient samples	oncogene	upregulated	enhancing SP1-mediated transcription of *CREB1*	increasing proliferation and decreasing apoptosis	[97]
*IUR*	Ph^+^ B-ALL patient samples and cell lines	tumor suppressor	downregulated	inhibiting STAT5-CD71 pathway	suppression of BCR-ABL1-mediated tumorigenesis	[98]
*CRNDE*	B-ALL cell lines and patient samples	oncogene	upregulated	sequestering miR-345-5p to upregulate *CREB*	promoting cell proliferation and inhibiting apoptosis	[99]
*LAMP5-AS1*	MLL rearranged B-ALL patient samples and cell lines	oncogene	upregulated	promoting methyltransferase activity of DOT1L to facilitate H3K9me2/me3 and increase HOXA expression	increasing colony formation and inhibiting differentiation	[100]
*TEX41*	B-ALL patient samples and cell lines	oncogene	upregulated	decreasing *CDK4*, *CDK6* and *p27* level, increasing *p21* and *p53* level	promoting cell growth and inhibiting cell cycle	[101]
*BALR-2*	B-ALL patient samples and cell lines	oncogene	upregulated	decreasing JUN and BIM expression	increasing cell growth and conferring resistance to prednisone treatment	[102]
*DUXAP8*	B-ALL patient samples and cell lines	oncogene	upregulated	sequestering miR-29a to increase *PIK3CA* expression	boosting proliferation, inhibiting apoptosis, and conferring Dox resistance	[103]

Abbreviations: *PVT1*, plasmacytoma variant translocation 1; *CASC15*, cancer susceptibility candidate 15; *ZEB1-AS1*, *ZEB1* antisense RNA 1; *BALR-6*, B-ALL-associated long RNA-6; *IUR*, imatinib upregulated; *CRNDE*, colorectal neoplasia differentially expressed; *LAMP5-AS1*, *LAMP5* antisense RNA1; *TEX41*, testis expressed 41; *BALR-2*, B-ALL-associated long RNA-2; *DUXAP8*, double homeobox A pseudogene 8.

**Table 3:** List of lncRNAs implicated in ALL (B- or T-subtype not differentiated) pathogenesis and treatment.

lncRNA	Subject	Function	Alteration	Target Regulation	Effect on ALL Cell Phenotype	References
*LINC00265*	ALL patient samples and cell lines	oncogene	upregulated	sponging miR-4500 to enhance *STAT3* expression	facilitating cell growth, proliferation, and migration	[104]
*PPM1A-AS*	ALL patient samples and cell lines	oncogene	upregulated	increasing phosphorylation of STAT3, Akt, and Notch4	promoting cell proliferation and inhibiting apoptosis	[105]
*TUG1*	Ph^-^ ALL patient samples	oncogene	upregulated	unknown	promoting CNS infiltration	[106]
*LINC00665*	ALL patient samples and cell lines	oncogene	upregulated	suppressing miR-101 to activate PI3K/Akt pathway	promoting cell viability, migration, and invasion	[107]
*SLCO4A1-AS1*	ALL patient samples and cell lines	oncogene	upregulated	sponging miR-876-3p to upregulate *RBBP6* and activate JNK	inhibited cell proliferation and promoted apoptosis	[108]
*PVT1*	ALL patient samples and cell lines	oncogene	upregulated	sponging miR-486-5p to increase *MAML3* expression; increasing *NOP2* and *c-Myc*, decreasing *p15*, *p16*	increasing cell viability restraining apoptosis; deregulating cell cycle, inhibiting apoptosis	[109,110]
*EBLN3P*	ALL patient samples and cell lines	oncogene	upregulated	negatively regulating miR-655-3p	promoting cell proliferation, invasion, and migration	[111]
*PINT*	ALL patient samples and cell lines	tumor suppressor	downregulated	increasing transcription of HMOX1	inducing apoptosis and causing cell cycle arrest	[112]
*HOTAIR*	ALL patient samples	oncogene	upregulated	increasing EZH2, LSD1, DNMT3A and DNMT3B level	promoting cell proliferation, survival, and migration	[113]
*HOXA-AS2*	ALL cell lines and patient samples	oncogene	upregulated	enhancing GC resistance via promoting HOXA3 expression	promotion of cell proliferation, inhibition of apoptosis	[114]
*SNHG16*	ALL cell lines and patient samples	oncogene	upregulated	sponging miR-124-3p	promoting ALL cell proliferation and migration	[115]
*MALAT1*	ALL samples and cell lines	oncogene	upregulated	sponging miR-205 to increase PTK7 expression	promoting cell proliferation and apoptosis	[116]

Abbreviations: *LINC00265*, long intergenic non-protein coding RNA 265; *PPM1A-AS*, protein phosphatase 1A antisense RNA; *TUG1*, Taurine upregulated gene 1; *LINC00665*, long intergenic non-protein coding RNA 665; *SLCO4A1-AS1*, *SLCO4A1* antisense RNA; EBLN3P, endogenous Bornavirus-like nucleoprotein; PINT, p53-induced transcript; HOTAIR, HOX transcript antisense intergenic RNA; *HOXA-AS2*, HOXA cluster antisense RNA2; *SNHG16*, small nuclear RNA host gene 16; MALAT1, metastasis associated lung adenocarcinoma transcript 1.

## Abbreviations

ALL	acute lymphoblastic leukemia
Allo-SCT	allogenic stem cell transplantation
ASOs	antisense oligonucleotides
BM	bone marrow
CART	chimeric antigen T-cell therapy
CNS	central nervous system
CR	complete remission
circRNA	circular RNA
DFS	disease-free survival
ENCODE	encyclopedia of DNA elements
ETP-ALL	early T-cell precursor ALL
GC	glucocorticoid
lncRNA	long non-coding RNA
MRD	minimal residual disease
MLL	mixed lineage leukemia gene
ncRNA	non-coding RNA
nt	nucleotide
OOCS	orientation-opposite complementary sequences
ORFs	open reading frames
Pol II	RNA polymerase II
PRC	polycome repressive complex
RBPs	RNA binding proteins
RNA-seq	RNA-sequencing
SNP	Single nucleotide polymorphism
TKIs	tyrosine kinase inhibitors
TAL1	T-cell acute lymphocytic leukemia protein 1
TLX1	T-Cell Leukemia Homeobox 1
